# Cognitive reappraisal and nasal cytokine production following experimental rhinovirus infection

**DOI:** 10.1016/j.bbih.2019.100012

**Published:** 2019-11-14

**Authors:** Ryan L. Brown, Anoushka D. Shahane, Michelle A. Chen, Christopher P. Fagundes

**Affiliations:** a Rice University, 6100 Main Street, Houston, TX, 77005, United States; b The University of Texas MD Anderson Cancer Center, 1515 Holcombe Boulevard, Houston, TX, 77030, United States; c Baylor College of Medicine, 1 Baylor Plaza, Houston, TX, 77030, United States

## Abstract

Following exposure to the common cold (i.e., rhinovirus), locally produced nasal cytokines (rather than the infection itself) drive the progression of one’s symptoms (Hendley et al., 1973; Cohen et al., 1999). Stress-induced local inflammation exacerbates local cytokine production (e.g., marital hostility; Kiecolt-Glaser et al., 2005). An individual’s ability to effectively manage their emotions is a critical component of positive health and well-being. Here, we evaluated whether one’s self-reported frequency of cognitive reappraisal, an adaptive emotion regulation strategy, predicts nasal cytokine production following experimental rhinovirus exposure. Emotion regulation strategies were assessed at baseline prior to experimental infection. After the baseline assessment, each participant was exposed to a strain of rhinovirus (RV-39) and followed for 5 days in quarantine. Nasal interleukin (IL)-1β, IL-6, and IL-8 and subjective symptoms were assessed at baseline and on each of the 5 days of quarantine. A multilevel analysis of the data for 159 participants with documented infection demonstrated that less frequent use of cognitive reappraisal predicted heightened production of the nasal cytokine composite. Those who self-reported using cognitive reappraisal strategies less frequently displayed elevated nasal IL-6 and IL-8. Among the 63 participants with clinical cold, less frequent use of cognitive reappraisal was associated with heightened production of nasal IL-1β, IL-6, and IL-8. In ancillary analyses, the composite of nasal cytokines was associated with the severity of one’s subjective symptoms across the 5 days. Findings suggest that emotion regulation strategies, particularly cognitive reappraisal, influence illness trajectories during rhinovirus infection.

## Highlights

• Greater use of cognitive reappraisal predicted reduced nasal IL-6 production.
• Greater nasal cytokine production predicted more severe symptoms.
• Emotion regulation strategies influence illness trajectories during a cold.

Following exposure to the common cold (i.e., rhinovirus), locally produced nasal cytokines (rather than the infection itself) drive the progression of one’s symptoms (Hendley et al., 1973; Cohen et al., 1999). There is mounting evidence that psychological elements (e.g., loneliness; LeRoy et al., 2017) influence one’s subjective symptom experience during rhinovirus infection. Stress-induced local inflammation exacerbates local cytokine production (e.g., marital hostility; Kiecolt-Glaser et al., 2005), which suggests that local cytokine production is likely the mechanism through which psychological stress exacerbates subjective symptoms when infected by rhinovirus. In this investigation, we explore whether individual differences in one’s ability to regulate emotions affect the timing and magnitude of nasal cytokine production.

Emotions provide essential feedback for how to react to our environment. Despite the usefulness of emotions, people often experience emotions that negatively impact their well-being. In these circumstances, it is advantageous to alter the emotion itself or the intensity/duration of the emotion. An individual’s tendency to effectively manage their emotions is a critical component of mental health. Indeed, those who are better at managing their emotions have better physical and mental health outcomes (Aldao et al., 2010; Florin et al., 1985; Greer and Watson, 1985; Hu et al., 2014; Verzeletti et al., 2016).

The way people adapt their emotions to their environment, perhaps changing which emotions they feel, or suppressing (rather than experiencing) their emotions entirely, are instances of emotion regulation (Gross, 1998). The emotion regulation process model distinguishes between regulatory strategies employed before or during emotion generation and strategies which occur after one experiences an emotional response (Gross, 1998, 2002). *Cognitive reappraisal* occurs before or during emotion generation and involves modifying one’s appraisal of a situation to either increase or decrease negative or positive emotions (Gross, 1998; Samson and Gross, 2012). Conversely, *expressive suppression* occurs after emotion generation. In expressive suppression, one inhibits their ongoing negative or positive emotion-expression behavior (Gross, 2014). Individuals who use reappraisal habitually demonstrate better health outcomes, such as the reduced risk of cardiovascular disease-related inflammation, premature aging, and fewer depressive symptoms (Appleton et al., 2014; English et al., 2012; Gross and John, 2003; Verzeletti et al., 2016). Additionally, individuals who frequently use reappraisal report lower stress-related symptoms relative to those who use suppression (Moore et al., 2008). However, we do not yet know if one’s emotion regulation strategies affect local inflammatory outcomes during normal rhinovirus infection.

Following rhinovirus exposure, nasal cytokine production promotes one’s symptom experience; thus, nasal cytokines represent one mechanistic pathway connecting psychosocial factors to illness (Hendley et al., 1973; Cohen et al., 1999). Those who report more experiences of psychological stress also experience increased rates of clinical illness from various types of rhinovirus (Cohen et al., 1991). The relationship between psychological stress and clinical colds was primarily attributable to increased rates of infection among individuals reporting greater stress. Cohen et al. (1998) identified that chronic stressors (i.e., one month or longer) drive this effect because those who reported experiencing acutely stressful life events for less than a month are not at heightened risk for developing a cold. Cohen et al. (1999) further investigated the biological mechanisms through which psychosocial factors modify illness expression, identifying that local, nasal IL-6 production explained the relationship between perceived stress and illness (as assessed by mucous weight and symptomology). Indeed, nasal cytokines mechanistically connect psychosocial factors to illness expression.

The present study examined how individual differences in *regulating* one’s emotions might attenuate the effect of rhinovirus exposure on one’s nasal immune response. The host immune response is responsible for the majority of symptoms one experiences when infected with rhinovirus (Hendley et al., 1973). To our knowledge, no studies have examined the influence of specific emotion *regulation* strategies on nasal cytokine production.

## 1 The current study

Given the timing of when emotion regulation strategies occur, cognitive reappraisal might support control of affective responses during illness. Because cognitive reappraisal is associated with decreased systemic inflammation (Appleton et al., 2014), and psychosocial factors can also impair local inflammatory processes (e.g., wound healing; Kiecolt-Glaser et al., 2005), we utilized data collected from the Pittsburgh Cold Study to investigate the influence of cognitive reappraisal on local inflammation among those infected and/or clinically ill from rhinovirus exposure. This question was novel, as *local* inflammation has not yet been investigated as a mechanism through which emotion regulatory strategies affect health outcomes. Local inflammation is responsible for the symptoms associated with the common cold; thus, the present study aims to predict differences in biological mediators of illness expression based on one’s frequency of cognitive reappraisal.

Here, we propose that the antecedent-focused emotion regulation strategy of cognitive reappraisal will be negatively associated with nasal inflammation among (a) those infected with rhinovirus and, (b) those who meet clinical criteria for a cold. Instead of focusing on susceptibility as an outcome, we will use the biological mediators of illness expression as an outcome to identify whether endorsing more frequent use of cognitive reappraisal will predict attenuated nasal inflammation following experimental exposure to rhinovirus (RV)-39. Thus, our initial hypotheses are as follows: more frequent self-reported use of cognitive reappraisal will be associated with lower levels of nasal inflammation (as measured by a composite of IL-6, IL-8, and IL-1β) throughout the course of infection among both those infected (H1) and those with a clinical cold (H2).

### 1.1 Materials and methods

#### 1.1.1 Participants and procedure

The present study is a secondary data analysis utilizing data from The Pittsburgh Cold Study 3, a prospective viral challenge study that ran between 2007 and 2011. These data were collected by the Laboratory for the Study of Stress, Immunity, and Disease at 10.13039/100008047Carnegie Mellon University under the directorship of Sheldon Cohen, Ph.D.; and were accessed via the Common Cold Project website (www.commoncoldproject.com; grant number 10.13039/100008460NCCIH AT006694). In this project, 123 men and 90 women were recruited in Pittsburgh, Pennsylvania, via newspaper advertisements. Before beginning the study, volunteers completed a telephone screening interview and an in-person physical health evaluation with a study physician. Only those volunteers with viral-specific antibody titers ≤4 were eligible to participate to maximize the rate of infection. Prior to infection, participants completed baseline assessments including psychosocial questionnaires and biological assessments. Then, they were followed in quarantine for 5 days while being monitored for signs of illness and objective signs of illness. Finally, blood was collected for serological testing 28 days after virus exposure. All participants who completed the study received $1000 for their time and dedication.

#### 1.1.2 Assessment of infection and clinical cold

Infection was determined through evidence of viral shedding and/or changes in serum specific antibody titer as described by Gwaltney et al. (2009). Thus, participants were classified as being infected if researchers (a) recovered the challenge virus from the participant’s nasal secretions post-challenge or if (b) participants displayed a 4-fold or greater increase in the virus-specific antibody titer between the pre-viral challenge baseline and 28 days post-challenge.

Among those who were infected, some also met criteria to have *objectively* developed a clinical cold using criteria put forth by Cohen et al. (1997). Specifically, participants were identified as having a clinical cold if (a) their total baseline-adjusted mucus weight (i.e., summed across all days post-challenge) weighed 10 kg or more, or (b) if their average baseline-adjusted nasal mucociliary clearance time was 7 min or longer.

#### 1.1.3 Nasal inflammation

Nasal wash fluid was assayed (in duplicate) for interleukin (IL)-1β, IL-5 (PCS3 only), IL-6, IL-8, IL-10, tumor necrosis factor (TNF)-α, and interferon (IFN)-α using commercially available enzyme-linked immunosorbent assays (ELISAs; Endogen). Levels were converted to concentrations by correcting for dilution. There, the dilution of nasal secretion in the aspirate/wash is estimated by measurement of the urea concentration using a coupled enzyme reaction involving urease and glutamate dehydrogenase (Sigma Diagnostics Kit No. 66-UV, Sigma Chemical Co.). Briefly, 20 μl of aspirate/wash is added to 300 μl BUN (Endpoint) reagent at room temperature and the absorbance after 5 min is measured at 340 nm on a spectrophotometer. The validity of each run is assessed by the inclusion of a diluent blank and a urea standard (Sigma). The dilution factor in an aspirate/wash is calculated by dividing the urea concentration in mg/dl into the assumed normal blood urea concentration of 10 mg/dl. Here, we only test associations with IL-6, IL-8, and IL-1β because these are produced during the ill period of viral upper respiratory illnesses and their presence causes the majority of the symptoms individuals feel when sick with the common cold (Gentile et al., 2003; Hendley et al., 1973).

#### 1.1.4 Cognitive reappraisal

The 10-item Emotion Regulation Questionnaire was used to measure one’s tendency to utilize two cognitive strategies to regulate emotions, namely cognitive reappraisal (e.g., “I control my emotions by changing the way I think about the situation I’m in”) and expressive suppression (e.g., “I keep my emotions to myself.“) (Gross and John, 2003). Participants endorsed each scale item using a Likert scale ranging from 1 (strongly disagree) to 7 (strongly agree). Scores from each subscale (6-items for cognitive reappraisal; 4 items for expressive suppression) were summed to derive a total score in which higher scores indicated greater use of the respective strategy (Cronbach’s α  = 0.79). In the present study, we analyze the cognitive reappraisal subscale assessed at one time point before rhinovirus exposure.

#### 1.1.5 Cold symptoms

The cold-related symptoms in the Daily Symptom Scale were used to assess the number of cold symptoms present in participants during the experiment. The Daily Symptom scale had 8 items for each symptom (nasal congestion, sneezing, runny nose, sore throat, cough, headache, chills, or feeling under the weather). Participants rated the severity of their experience in the past 24 h on a 5 point Likert scale ranging from 0 (none) to 4 (very severe) for each item. The item scores were summed to create a Jackson Symptom Score (Jackson et al., 1958). Each Jackson Symptom Score was adjusted by subtracting the day’s score from the baseline score, as the scale is designed to measure changes in symptoms. The total adjusted daily cold symptoms, also known as the adjusted Jackson Symptoms Scores, were calculated by adding the adjusted values of the 8 Jackson symptoms within each post-challenge quarantine day. This scale has been utilized in previous investigations of viral symptoms (Cohen et al., 1997; Gwaltney et al., 1980; LeRoy et al., 2017).

#### 1.1.6 Other covariates

We also included several variables related to illness expression. Age, sex, and educational attainment were assessed via self-report. Age was entered as a continuous variable. Sex was binary such that 0 = male and 1 = female. Education was coded categorically with 9 levels where 1 = did not finish high school and 9 = Ph.D., M.D., or another higher degree. Season and day of the trial were tracked throughout the study. Body mass index (BMI) was calculated at two time points, once between 3 and 21 days before the cold challenge and once 28 days following exposure. Thus, BMI here represents the participant’s average BMI across the sessions before and after this cold challenge.

### 1.2 Statistical analysis

All analyses were conducted in *R* (version 1.1.456; R Core Team, 2019). Analyses relied on the *nlme* package (Pinheiro et al., 2019) and the *ggplot2* package for data visualization (Wickham, 2016). We also used the *pacman* and *apaTables* packages to generate the tables presented here (R Core Team, 2019; Stanley, 2018).

#### 1.2.1 Data preprocessing

Preliminary statistical analysis included assessment of normality of distributions and examination for skewness and kurtosis. The nasal inflammatory markers were skewed as is normally expected for inflammatory markers (Shields et al., 2016). Thus, we normalized each marker using a (base 10) log transformation before analysis. There were very little missing data in this dataset; thus, we only included individuals without missing data for any of the inflammatory markers (*n* = 212). We examined the residuals following each analysis to ensure they did not appear to deviate meaningfully from a normal distribution.

Next, in order to control for type I error, we created a composite index of all proinflammatory nasal cytokines previously identified to be produced during the ill period of an upper respiratory infection, and previously tied to one’s illness expression. Specifically, this composite index included IL-6, IL-1β, and IL-8. For each cytokine, we calculated z-scores from the (base 10) log-transformed values. These values were then averaged to produce a summary nasal inflammation construct from Day 1 to Day 5 following rhinovirus exposure. This established method allows researchers to analyze multiple correlated dependent variables and is also useful when examining immune markers that function similarly (Dattalo, 2013; Fagundes et al., 2012). Indeed, because these proinflammatory markers work together in vivo, this composite index reflects a coordinated immune response. In the primary analyses, we tested for associations between cognitive reappraisal where the nasal cytokine composite was the outcome of interest among (1) those who were infected, and then (2) those who met objective clinical criteria for a cold. We also ran separate models to assess the association between cognitive reappraisal and each individual nasal cytokine of interest within those two samples. In order to control for baseline nasal inflammation, we computed *z*-scores of the (base 10) log-transformed values for each nasal cytokine of interest (i.e., IL-6, IL-8, IL-1β). This Day 0 nasal inflammation composite was included in each model, along with age, sex, BMI, educational attainment, the season of the trial, and the day of the trial.

#### 1.2.2 Fitting the model

##### 1.2.2.1 Testing for intercept variability

First, we tested whether there was statistically significant variability in the intercepts across groups. Here, the level 2 grouping variable was the person. Thus, we investigated whether there was significant variability in intercepts across people by first estimating an unconditional means model, which contains only the random intercept variance term to allow the intercepts (means) to differ across individuals. The null model partitions total variance within a dependent variable into the within- and between-persons components. Here, the intercept for each null model represents the mean level of that variable across individuals. A substantial proportion (57%) of the variance in the nasal cytokine composite was within-individuals for those who were infected. For those who met clinical criteria for a cold, 45% of the variance in the nasal cytokine composite was within-individuals.

Additionally, −2 log likelihood results indicated that the model including the random effect of time fit the data better than the model without that random effect. Akaike information criterion (AIC) was used to assess the relative quality between the models. AIC was necessary to compare the models in order to investigate to what extent information is lost in each model; the more lost, the lower quality the model (Bozdogan, 1987; Vrieze, 2012). Bayesian information criterion (BIC) was also computed as estimates of posterior probability (Vrieze, 2012). The lower the AIC and BIC values, the closer the model is to the truth. The standard −2 log likelihood was used, [-2log*L ​+ ​kp*], where *L* is the likelihood function, *p* is the number of parameters in the model, *k* is 2 for AIC, and *log(n)* is for BIC.

##### 1.2.2.2 Investigating sources of variance

Before testing linkages in the hypothesized model, we further investigated whether there were systematic within- and between-individual variance that existed in the dependent variable (proinflammatory cytokine composite). We examined the contextual effect of one’s baseline levels of nasal cytokines. A model including baseline nasal cytokines explained 57% more variance in the intercepts than a model not including one’s baseline nasal inflammation before rhinovirus exposure. Thus, the variance in the intercepts between individuals was partially related to one’s baseline nasal inflammation.

##### 1.2.2.3 The relationship between time and nasal cytokines

Our next step was to delineate the nature of the day of infection’s influence on the nasal cytokine composite. We modeled the relationship between the day of infection and the nasal cytokine composite. There was a positive, linear relationship between day of infection and the nasal cytokine composite among those infected, *t*(631) = 3.00, *p* = .003, as well as among those who met clinical criteria for the cold, *t*(251) = 3.50, *p* < .001. We identified that for those infected, but not those who meet clinical criteria for the cold, a model that allows the slope between the day of infection and nasal cytokine composite to randomly vary fits the data better than a model that fixes the slope to a constant value for all individuals (*p*_*infected*_ < .001).

## 2 Results

### 2.1 Description of the sample

Of the 212 number of participants who had complete data for the variables of interest, 159 (75%) were infected and 63 (30%) developed clinical colds. Descriptive statistics and correlations between key variables can be found in Table 1.

**Table 1:** Means, standard deviations, and correlations with confidence intervals for key study variables in the infected sample.

Variable	*M*	*SD*	1	2	3	4	5	6	7	8	9
1. Age	30.17	11.09									

2. BMI	27.54	6.48	.34**								
			[.31, .37]								

3. Sex	0.41	0.49	.05**	.14**							
			[.01, .08]	[.11, .17]							

4. Educational Attainment	5.22	1.73	.09**	-.10**	.08**						
			[.06, .12]	[-.13, −.07]	[.05, .12]						

5. Season of the Trial	2.20	0.79	.01	-.21**	-.04*	.07**					
			[-.03, .04]	[-.24, −.18]	[-.07, −.01]	[.03, .10]					

6. Cognitive Reappraisal	29.17	6.83	.11**	-.03	.01	.06**	-.07**				
			[.08, .14]	[-.06, .00]	[-.02, .04]	[.02, .09]	[-.10, −.04]				

7. Expressive Suppression	13.62	4.87	.09**	-.09**	-.27**	.00	.01	.04*			
			[.06, .12]	[-.12, −.06]	[-.30, −.24]	[-.03, .03]	[-.02, .04]	[.00, .07]			

8. Baseline Nasal Cytokine Composite	−0.00	0.79	-.16**	.06**	-.02	.11**	-.17**	.02	-.02		
			[-.19, −.13]	[.03, .10]	[-.05, .01]	[.08, .14]	[-.20, −.14]	[-.01, .05]	[-.05, .01]		

9. Nasal Cytokine Composite	0.13	0.79	-.19**	.05**	.02	.10**	.05**	-.18**	-.06**	.43**	
			[-.22, −.16]	[.02, .08]	[-.02, .05]	[.06, .13]	[.02, .08]	[-.21, −.15]	[-.09, −.03]	[.40, .45]	

10. Symptoms	2.93	3.41	-.01	.08**	.17**	-.04*	.09**	-.02	-.07**	.06**	.18**
			[-.04, .02]	[.05, .12]	[.14, .20]	[-.07, −.01]	[.06, .12]	[-.05, .01]	[-.10, −.04]	[.03, .09]	[.15, .21]

*Note.* The nasal cytokine composite consists of IL-6, IL-1β, and IL-8. Sex was coded as 0 = Male, 1 = Female. Education was coded categorically with 9 levels where 1 = did not finish high school and 9 = Ph.D., MD, or another higher degree. *M* and *SD* are used to represent mean and standard deviation, respectively. Values in square brackets indicate the 95% confidence interval for each correlation. The confidence interval is a plausible range of population correlations that could have caused the sample correlation (Cumming, 2014). * indicates *p* ​< ​.05. ** indicates *p* < .01.

### 2.2 Full composite model

**Fig. 1:**
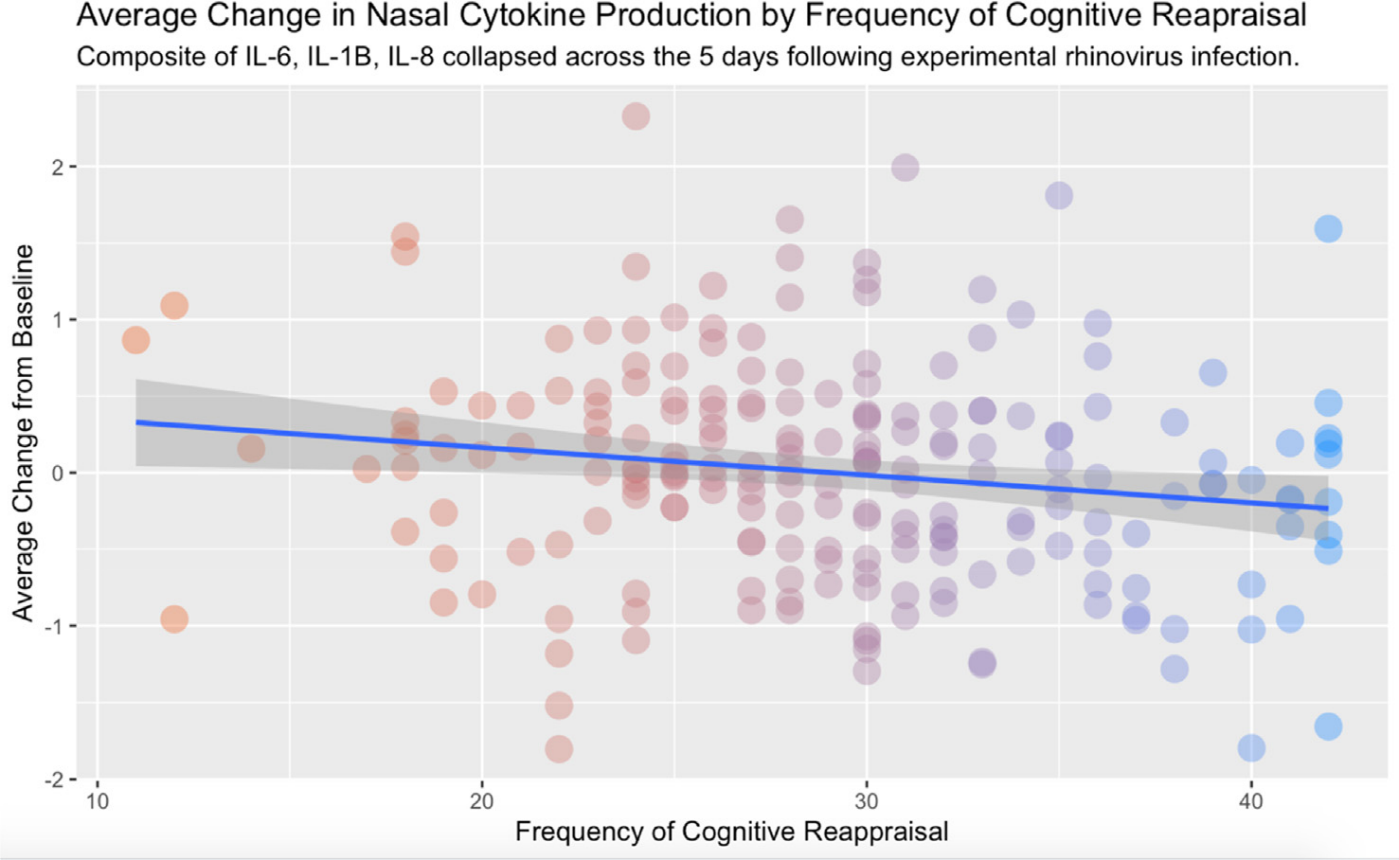
Average change in nasal cytokine production from baseline for those infected with rhinovirus based on self-reported frequency of cognitive reappraisal.

We first considered the unadjusted (i.e., correlational) relationship between one’s frequency of self-reported cognitive reappraisal and nasal cytokine production following rhinovirus exposure. Among participants who were infected, less frequently reported use of cognitive reappraisal strategies was negatively associated with the nasal cytokine composite (*r(*157*)* = -.18, *p* < .01; see Fig. 1).

The critical analysis tested the association of cognitive reappraisal with the nasal cytokine composite, controlling for baseline nasal inflammation, age, sex, BMI, day of trial, educational attainment, and the season of trial. We compared two different full composite models, which varied by their fixed and random effects. In the first model, we tested linear fixed effects and linear random effects of time. In the second model, we tested linear fixed effects and no random effects of time. AIC and BIC values were computed for each of these models to assess model fit. Table 2 contains AIC and BIC values for each model. The AIC selected the first model (linear fixed effects and linear random effects) as the best fit, and the BIC selected the second model (linear fixed effects and no random effects) as the best fit.

**Table 2:** Akaike’s information criterion and Bayesian information criterion for each model.

Model	Fixed Effects	Random Effects	AIC	BIC
Model 1	Linear	Linear	1422.8	1488.2
Model 2	Linear	None	1426.3	1482.4

**Fig. 2:**
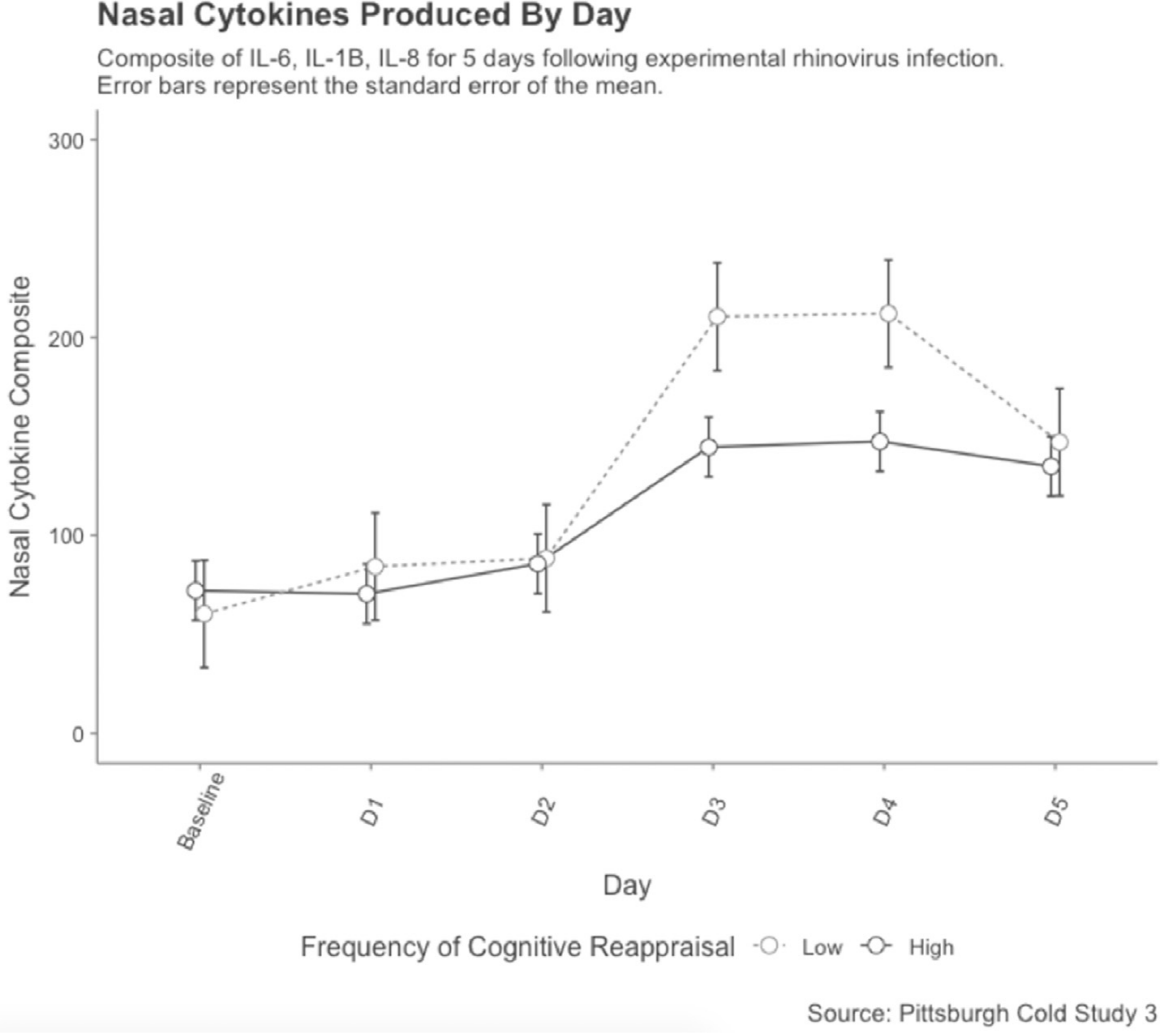
Nasal cytokines produced by day.

In the full model, those who self-reported using cognitive reappraisal strategies less frequently displayed elevated nasal inflammation in response to the cold challenge (*b* = −0.02, *95% CI* [-0.03, −0.01], *p* = .002; see Table 3). Among those who met clinical criteria for the cold, less frequent use of cognitive reappraisal strategies was also associated with elevated nasal inflammation (*b* = −0.04, *95% CI* [-0.06, −0.02], *p* < .001; see Table 4). See Fig. 2 for a graphical depiction of the changes observed in the nasal cytokine composite during the 5-day quarantine based on one’s frequency of cognitive reappraisal.

**Table 3:** Nasal inflammation in the infected sample.

	Full Composite Model		IL-6		IL-8		IL-1b	
Predictors	Estimates	CI	Estimates	CI	Estimates	CI	Estimates	CI
Intercept	0.12	−0.50 – 0.73	0.26	−1.17 – 1.70	2.49 ​***	1.53–3.46	−0.06	−1.44 – 1.32
Baseline Nasal Composite	0.44 ​***	0.34–0.54						
Age	−0.01 ​**	−0.02–−0.00	−0.02 ​*	−0.04–−0.00	−0.01 ​*	−0.02–−0.00	−0.02 ​*	−0.04–−0.00
Day of Trial	0.04 ​*	0.01–0.07	0.48 ​***	0.40–0.57	0.23 ​***	0.17–0.28	0.23 ​***	0.16–0.29
Gender	0.01	−0.15 – 0.17	0.09	−0.28 – 0.46	−0.07	−0.30 – 0.15	0.09	−0.26 – 0.45
Educational Attainment	0.03	−0.01 – 0.08	0.08	−0.02 – 0.19	0.06	−0.01 – 0.12	0.03	−0.07 – 0.13
Body Mass Index	0.01	−0.00 – 0.03	0.02	−0.01 – 0.05	0.02	−0.00 – 0.04	0.03	−0.00 – 0.06
Season of Trial	0.10	−0.00 – 0.20	0.13	−0.11 – 0.37	0.09	−0.05 – 0.23	0.28 ​*	0.05–0.50
Cognitive Reappraisal	−0.02 ​**	−0.03–−0.01	−0.03 ​*	−0.06–−0.01	−0.03 ​**	−0.04–−0.01	−0.02	−0.05 – 0.00
Baseline IL-6			0.36 ​***	0.21–0.51				
Baseline IL-8					0.51 ​***	0.41–0.61		
Baseline IL-1b							0.60 ​***	0.47–0.72
**Random Effects**								
σ^2^	0.34		1.99		0.96		1.37	
τ_00_	0.03 _subj_id_		0.05 _subj_id_		0.04 _subj_id_		0.26 _subj_id_	
τ_11_	0.00 _subj_id.Day.n_		0.06 _subj_id.Day.n_		0.01 _subj_id.Day.n_		0.02 _subj_id.Day.n_	
ρ_01_	0.82		0.88		0.75		0.89	
ICC	0.29		0.34		0.21		0.38	
N	159 _subj_id_		159 _subj_id_		159 _subj_id_		159 _subj_id_	
Observations	791		793		792		792	
Marginal R^2^/Conditional R^2^	0.265/0.476		0.213/0.480		0.321/0.466		0.306/0.573	

*p ​< ​.05 ​ ​ ​**p ​< ​.01 ​ ​ ​***p ​< ​.001.

**Table 4:** Nasal inflammation in the clinical cold sample.

	Full Composite Model		IL-6		IL-8		IL-1b	
Predictors	Estimates	CI	Estimates	CI	Estimates	CI	Estimates	CI
Intercept	1.68 ​**	0.48–2.87	4.01 ​**	1.61–6.40	4.05 ​***	2.14–5.96	2.97	−0.04 – 5.99
Baseline Nasal Composite	0.30 ​***	0.13–0.47						
Age	−0.01 ​*	−0.02–−0.00	−0.02 ​*	−0.05–−0.00	−0.01	−0.03 – 0.00	−0.02	−0.05 – 0.01
Day of Trial	0.08 ​**	0.03–0.13	0.59 ​***	0.47–0.72	0.30 ​***	0.21–0.39	0.27 ​***	0.17–0.37
Gender	−0.09	−0.36 – 0.17	−0.10	−0.62 – 0.42	−0.01	−0.39 – 0.38	−0.33	−1.02 – 0.36
Educational Attainment	0.01	−0.07 – 0.08	0.02	−0.12 – 0.17	−0.00	−0.11 – 0.11	0.02	−0.17 – 0.20
Body Mass Index	−0.01	−0.03 – 0.01	−0.03	−0.07 – 0.01	−0.01	−0.04 – 0.02	0.00	−0.04 – 0.05
Season of Trial	0.03	−0.11 – 0.18	−0.01	−0.31 – 0.29	0.03	−0.18 – 0.25	0.14	−0.21 – 0.50
Cognitive Reappraisal	−0.04 ​***	−0.06–−0.02	−0.07 ​***	−0.11–−0.03	−0.03 ​*	−0.06–−0.00	−0.07 ​**	−0.12–−0.02
Baseline IL-6			0.24 ​*	0.06–0.42				
Baseline IL-8					0.45 ​***	0.30–0.60		
Baseline IL-1b							0.40 ​**	0.14–0.67
**Random Effects**								
σ^2^	0.31		2.05		1.20		1.30	
τ_00_	0.19 _subj_id_		0.04 _subj_id_		0.11 _subj_id_		1.03 _subj_id_	
τ_11_	0.01 _subj_id.Day.n_		0.04 _subj_id.Day.n_		0.01 _subj_id.Day.n_		0.03 _subj_id.Day.n_	
ρ_01_	−0.45		0.83		0.50		−0.15	
ICC	0.38		0.24		0.18		0.49	
N	63 _subj_id_		63 _subj_id_		63 _subj_id_		63 _subj_id_	
Observations	315		315		315		315	
Marginal R^2^/Conditional R^2^	0.246/0.529		0.303/0.470		0.285/0.413		0.238/0.610	

*p ​< ​.05 ​ ​ ​**p ​< ​.01 ​ ​ ​***p ​< ​.001.

### 2.3 Individual cytokines

In an unadjusted, correlational model among participants who were infected, less frequent use of cognitive reappraisal strategies were associated with lower IL-6 (*r(*157*)* = -.12, *p* < .01), lower IL-8 (*r(*157) = -.16, *p* < .01), and lower IL-1β (*r(*157) = -.13, *p* < .01). Those who self-reported using cognitive reappraisal strategies less frequently also displayed elevated nasal IL-6 (*b* = −0.03, *95% CI* [-0.06, −0.01], *p* = .016) and IL-8 (*b* = −0.03, *95% CI* [-0.04, −0.01], *p* = .001) in response to the cold challenge (see Table 3 for full results for infected sample). Those who self-reported using cognitive reappraisal strategies less frequently also displayed somewhat elevated nasal IL-1β in response to the cold challenge, although it was not statistically significant (*b* = −0.02, *95% CI* [-0.04, −0.00], *p* = .061). However, among those who met clinical criteria for the cold, less frequent use of cognitive reappraisal strategies was associated with significantly elevated nasal IL-1β (*b* = −0.07, *95% CI* [-0.11, −0.03], *p* = .007), IL-6 (*b* = −0.07, *95% CI* [-0.11, −0.03], *p* < .001), and IL-8 (*b* = −0.03, *95% CI* [-0.06, 0.00], *p* = .042). However, the confidence interval for IL-8 did include 0. Full results for the clinical cold sample can be found in Table 4.

### 2.4 Ancillary analyses

In post-hoc analyses, we tested whether reappraisal or suppression predicted who became infected. Self-reported use of cognitive reappraisal did not predict who became infected with rhinovirus (*p* = .26), nor did self-reported use of expressive suppression predict who became infected with rhinovirus (*p* = .83).

Next, we explored the relationship between frequency of expressive suppression and nasal inflammation among those either infected or who met clinical criteria for the cold. More frequent self-reported use of expressive suppression did not predict the nasal cytokine composite in the infected (*p* > .9), nor clinical cold samples (*b* = 0.02, *95% CI* [-0.01, 0.05], *p* = .13).

Finally, we extended our findings to identify whether cognitive reappraisal predicts one’s *symptoms* when infected with rhinovirus. Frequency of self-reported cognitive reappraisal did not predict cold symptoms (*p* > .4), nor did the frequency of self-reported expressive suppression in this sample (*p* > .6); however, elevated levels of the overall cytokine composite post-challenge predicted more severe self-reported cold symptoms (*b* = 3.66, *95% CI* [2.57, 4.76], *p* < .001), after adjusting for pre-challenge self-reported symptoms in addition to controlling for age, sex, BMI, educational attainment, and the season of trial.

## 3 Discussion

In this study, we identified that individuals who reported more frequently employing cognitive reappraisal experienced an attenuated local inflammatory response while infected with rhinovirus (H1). Indeed, baseline nasal inflammation, age, sex, BMI, day of trial, educational attainment, and the season of trial did not explain the association between cognitive reappraisal and attenuated local inflammatory responses. It is also notable that this finding was similar among both those who were infected and those who met clinical criteria for the cold (H2). Although neither emotion regulation strategy directly predicted symptoms, the overall nasal cytokine composite did reliably predict self-reported cold symptoms, which supports the notion that the production of proinflammatory cytokines drives one’s symptom experience (Gentile et al., 2003; Naclerio et al., 1988; Turner et al., 1998). These findings add to the burgeoning literature on psychosocial modifiers of illness expression.

To our knowledge, this is the first paper to demonstrate that individual differences in emotion regulation strategies influence inflammatory responses during the course of an infection. A major strength of this study was that the emotion regulation questionnaires were administered in a pre-challenge period before infection. Thus, one’s tendency to use cognitive reappraisal influenced local nasal inflammation during the rhinovirus infection, whether they utilized cognitive reappraisal strategies during the viral challenge or not. Across both, those infected and those meeting criteria for a clinical cold, greater self-reported frequency of cognitive reappraisal predicted attenuated nasal IL-6. The results for IL-8 and IL-1β were more mixed across those infected and meeting clinical criteria for the cold. Each of these cytokines are produced in response to rhinovirus infection. Previously, IL-6 has been established as an essential biological mediator in the relationship between psychological stress and illness expression following both influenza and rhinovirus infections (Cohen et al., 1999; Doyle et al., 2006).

Psychosocial elements also influence nasal cytokine production through the neuroendocrine system. Cortisol levels during infection were not collected. As a result, we were not able to directly test the association between emotion regulation strategies and cortisol levels during infection, prior work demonstrated that expressive suppression, but not cognitive reappraisal, predicted diurnal cortisol levels in naturalistic settings (Otto et al., 2018). We do not anticipate this association to be different during the course of infection; that is, we expect those who tend to utilize expressive suppression strategies more frequently to exhibit elevated levels of diurnal cortisol. However, rather than cortisol levels themselves, a recent study identified that the relationships between life stress, cortisol, and illness susceptibility/expression resulted from glucocorticoid receptor resistance such that chronic stress led to reduced sensitivity of leukocytes to the inhibitory effect of cortisol (i.e., glucocorticoid receptor resistance), which then predicted one’s risk for developing a cold as well as the number of proinflammatory nasal cytokines produced (Cohen et al., 2012). For the purposes of predicting one’s symptom experience, cortisol levels were less useful than glucocorticoid receptor resistance (Cohen et al., 2002, 2012). Future work ought to examine the relative influence of glucocorticoid receptor resistance contributing to increased production of nasal cytokines during illness.

There was no relationship between expressive suppression and nasal cytokine production among those in either sample. We investigated suppression to see if there would be an inverse pattern relative to that seen with cognitive reappraisal. Given that there is no negative correlation between expressive suppression and cognitive reappraisal and that they are independent constructs (Lopez and Denny, 2019; Moore et al., 2008), this finding is sound.

Rhinovirus symptoms generally peak between 1 and 3 days; thus, knowing that this timing might differ across individuals, we modeled the effect of time. We quantitatively compared the models to determine which model was the best fit (i.e., using Akaike’s information criterion [AIC] and Bayes information criterion [BIC]). The AIC values suggest the first model testing the association of cognitive reappraisal with the nasal cytokine composite, which included linear effects for both the fixed and random effects, was the best fit. The BIC values suggest the second model testing linear effects for the fixed effects and no effects for the random effects was the best fit. In the present study, AIC and BIC values disagree in selecting the best fit model. AIC and BIC differ in the way they penalize model complexity. Specifically, AIC tends to favor more complex models (linear effects for both fixed effects and random effects), while BIC penalizes more complex models more heavily and prefers simpler models (linear effects only for the fixed effects) (Vrieze, 2012). If one’s goal is to identify a strong predictive model, the AIC selected model should be favored, while if one’s goal is to identify a strong explanatory model, the BIC selected model should be favored (Shmueli, 2010). Given that the present study is more concerned with selecting a predictive model, the AIC selected model—the first model with linear fixed and random effects—was selected. Furthermore, Vrieze (2012) suggests that in studies with finite sample sizes (which is often the case in psychological research), the BIC typically performs worse than the AIC. As a result, it is reasonable that the AIC and BIC model selections differed in the present study, and perhaps the AIC model selection should be preferred. However, it would also be relevant to examine individual differences in the timing of nasal cytokine production and the onset of one’s symptoms.

Several future directions emerge from these findings. Cognitive reappraisal has the potential to be used in treatment settings as it is relatively inexpensive and easy to administer. For example, Troy and colleagues suggest that interventions that target negative appraisals could be particularly effective for depression (Troy et al., 2010). Another advantage of emotion regulation training is that it can be delivered directly to someone’s mobile device, which offers more flexibility and access than many other intervention methods. Notably, only one study has examined psychosocial modifiers of illness expression by examining nasal cytokine production in response to experimental rhinovirus exposure. Doyle, Gentile, and Cohen (2006) identified that a higher, positive emotional style was associated with lower nasal IL-6 levels and that nasal IL-6 mediated the relationship between positive emotional style and systemic and nasal symptom clusters. Specifically, high levels of dispositional positive emotional style were associated with fewer nasal, throat, and systemic symptoms and signs. Future work might examine the relative influence of emotional style versus emotion regulation strategy in the future.

The current study had some limitations that can be addressed in future studies. Cognitive reappraisal was only assessed at baseline and represented a self-reported frequency of cognitive reappraisal; future studies should investigate whether fluctuations in one’s frequency of cognitive reappraisal use also predicts nasal inflammation throughout the illness. The parent study to ours did not assess systemic inflammation and it would be worthwhile to examine potential differences between the impact of cognitive reappraisal on both local and systemic inflammation. The sample was largely healthy and middle-aged. Future investigations could also examine these associations across different stages of life (e.g., in the elderly). Studying these associations in older adults would also be important because elderly adults are more likely to have compromised immune systems (Miller, 1996).

Our findings suggest that emotion regulation strategies, particularly cognitive reappraisal, influence local nasal cytokine production during rhinovirus. There is reason to suspect that the effects reported here might *underestimate* the impact of cognitive reappraisal on nasal inflammation. Although the ERQ is the gold-standard for a self-report measure of emotion regulation, it will be important to examine differences in individuals’ *effectiveness* when utilizing these strategies. Given that the cognitive reappraisal subscale on the ERQ indexes cognitive reappraisal *frequency*, future work would need to further explore if *frequency* is a proxy for *ability*. This work can also be extended beyond the experience of the common cold to examine how rhinovirus infection affects asthma, a chronic obstructive pulmonary disorder, and those who are immunocompromised (Winther, 2011). Furthermore, this offers a promising avenue for intervention work because emotion regulation strategies can be *trained*; this training effect may be stronger or weaker than the effect of one’s ‘natural’ tendency to cognitively reappraise. In sum, we identified that cognitive reappraisal is an important factor in predicting nasal inflammation in response to rhinovirus infection above and beyond a host of demographic, seasonal, and biological factors previously associated with cytokine production.

## Declaration of competing interest

None.
